# Nonlinear damage creep model of concrete considering the influence of temperature and its parameter variation law analysis

**DOI:** 10.1371/journal.pone.0327314

**Published:** 2025-07-07

**Authors:** Lu Wang

**Affiliations:** School of Civil Engineering, Liaodong University, Dandong, China; Shandong University of Technology, CHINA

## Abstract

In order to study the creep characteristics of concrete under different temperatures, the uniaxial creep test of concrete is carried out. On this basis, the nonlinear creep model of concrete is established by using the fractal order derivative theory. According to the characteristics of the classical creep curve and the creep equation, the calculation method of creep parameters is determined. The variation law of each creep model parameter under different stress and temperature is analyzed. The results showed that the model can well reproduce the change trend of the curve during the creep process of concrete under different temperature conditions and stress levels, indicating that the model has strong adaptability and accuracy in capturing the creep law. In the later stage of concrete creep, the accelerated creep stage is often accompanied by complex situations such as rapid accumulation of internal damage and rapid deterioration of the structure. The traditional model has limitations in the accurate description of this stage, and the new model can effectively solve this problem. Undoubtedly, it enhances its value and advantages in practical applications. The elastic modulus of the elastomer controls the instantaneous strain. The elastic modulus, viscosity coefficient and fractal order of viscoelastic body control the viscoelastic creep and creep rate. Viscosity coefficient and fractal order of viscoplastic body control the viscoplastic creep and creep rate.

## 1. Introduction

As one of the most widely used building materials in the field of modern civil engineering, concrete plays a key role in the construction of various types of infrastructure, such as high-rise buildings, bridges, dams and underground structures [1–3]. However, concrete is by no means an ideal elastomer. In the case of long-term constant load, it will inevitably undergo deformation that gradually increases with time. This phenomenon is creep [4,5]. Creep not only affects the service performance of concrete structures, but also may cause structural cracks, deformation overrun, and even endanger the safety and durability of the overall structure [6,7]. It has become a key problem that has long been concerned by the engineering community and needs to be overcome. Traditional concrete creep research is mostly based on normal temperature conditions [8–10]. It is assumed that the material properties are constant, and the linear model is used to describe its creep behavior. Admittedly, this kind of simplified treatment has certain feasibility in some conventional engineering scenarios, and can give a rough deformation prediction, aided design and construction process. However, in actual working conditions, concrete structures are often exposed to complex and variable temperature environments [11,12]. Whether it is the extreme heat and cold in extreme climate, or the high temperature radiation around the industrial facilities, or the temperature gradient caused by the hydration heat of cement in the mass concrete, the temperature fluctuation is everywhere, which has a profound impact on the creep characteristics of concrete [13,14].

Domestic and foreign scholars have achieved fruitful results in the study of concrete creep. On the basis of MPS theory, Yu et al. [15] made some appropriate corrections. The relationship between long-term creep viscosity and micro-prestress was also redefined. At the same time, based on thermodynamics, the influence of temperature and humidity changes on the evolution of micro-prestress was re-set. The transient thermal creep effect was modified to be related to humidity. The results proved the correctness of the modification and the practicability of the improved model. Considering the influence of temperature change on creep, Zhao et al. [16] proposed an improved Kelvin creep model. The improved Kelvin model predicted the early tensile creep of concrete under variable temperature conditions. The Burgers segmented creep model proposed by Wu et al. [17] well describes the creep behavior of bio-based apricot shell concrete at low load levels. It is recommended to use this model to express the long-term creep behavior of bio-based apricot shell concrete in aqueous environments. Lv et al. [18]proposed the creep increase coefficient and shrinkage increase coefficient of adhesive mortar according to the different content of adhesive mortar, and established the shrinkage and creep model of recycled aggregate concrete. Compared with the experimental results, the model calculation results met the accuracy requirements. Liang et al. [19] developed an improved Maxwell model based on structural buffers to describe ultra-slow creep behavior. Pasalli et al. [20] proposed a concrete creep prediction model based on the modification of the B3 model. The material constants obtained from the short creep test were modified. The creep parameters obtained from the test can be better used to predict the creep of concrete.

The above studies seldom consider the influence of temperature on the creep of concrete structures. A method for determining creep parameters is also not proposed. Therefore, this paper will carry out the creep characteristics test of concrete structure under different temperature. On this basis, the nonlinear creep model of concrete is established by using the fractal order derivative theory. According to the characteristics of the classical creep curve and the creep equation, the calculation method of creep parameters is determined. The variation law of each creep model parameter under different stress and temperature is analyzed. Finally, the relationship between creep parameters and temperature and stress is substituted into the creep model to obtain a nonlinear creep model considering the influence of temperature and stress.

## 2. Creep test of concrete under different temperatures

### 2.1. Mechanical properties test of concrete

**Fig 1:**
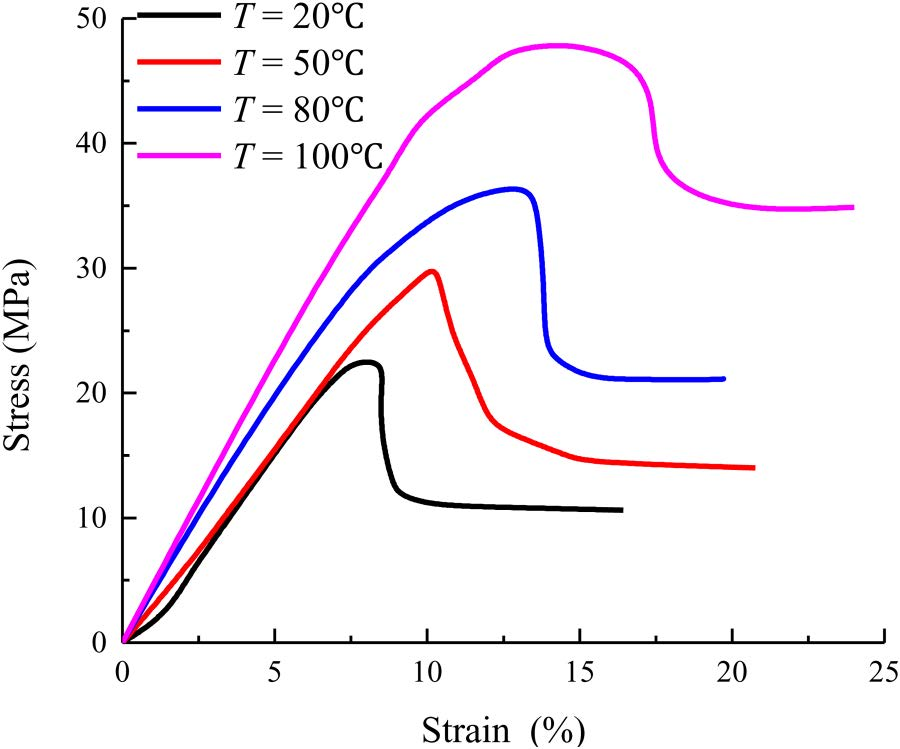
The stress-strain curves of concrete under different temperatures.

The stress-strain curves of concrete under different temperatures are shown in Fig 1.

It can be seen from Fig 1 that as the temperature increases, the free water inside the concrete begins to evaporate, and the cement paste gradually shrinks. It leads to micro cracks in the concrete. These micro cracks continue to expand, making the elastic modulus of concrete gradually reduced. The slope of the rising section of the stress-strain curve gradually becomes smaller. With the further increase of temperature, the peak stress of concrete decreases significantly. This is because the high temperature causes the cement hydration products inside the concrete to decompose and destroy, and the structure of the concrete gradually loosens. At the same time, the peak strain will increase with the increase of temperature. This is due to the increase of plastic deformation ability of concrete at high temperature, and the expansion and extension of internal micro cracks are more sufficient. The descending section of the stress-strain curve of concrete at high temperature is relatively gentle and has obvious residual strength. This is because although the high temperature causes the cement hydration products inside the concrete to be destroyed and the structure to be loose, the aggregate can still play a supporting role to a certain extent. It makes the concrete maintain a certain bearing capacity.

### 2.2. The creep characteristic curves of concrete

During the long-term creep tests, the temperature control system maintained an accuracy of ±0.5°C, with the standard deviation of temperature fluctuations not exceeding 0.4°C throughout the 30-day continuous testing period. The temperature variations resulted in less than 5% error in creep strain predictions, with the impact on the fractional-order parameter *α*vp remaining within 15% of its confidence interval width.

**Fig 2:**
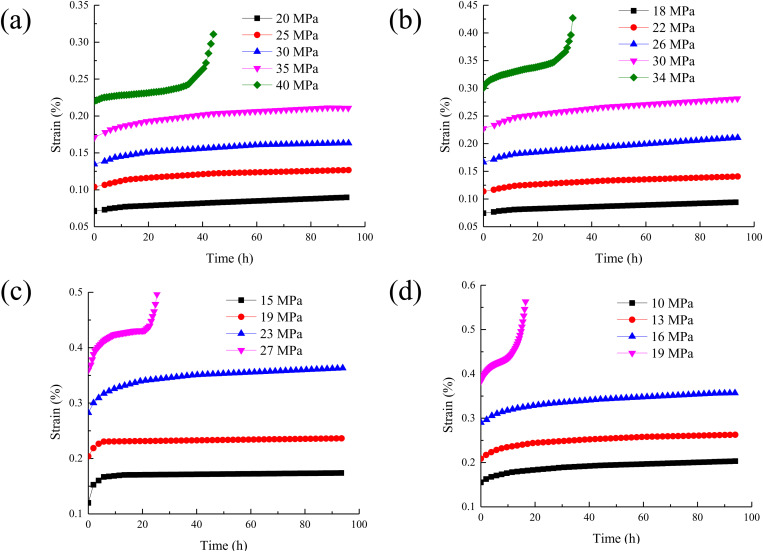
The creep characteristic curves of concrete under different temperatures. (a) T = 20 °C; (b) T = 50 °C; (c) T = 80 °C; (d) T = 100 °C.

According to the mechanical properties test of concrete, the creep characteristics test of concrete is carried out [21,22]. The creep characteristic curves of concrete under different temperatures are drawn as shown in Fig 2.

It can be seen from Fig 2 that the creep curves of concrete under different temperatures are basically the same. At normal temperature, the creep of concrete is mainly caused by the viscous flow of gel particles in cement paste and the slow development of internal micro cracks. With the increase of temperature, the instantaneous strain of concrete is also increasing. This is due to the increase of temperature, which leads to the increase of molecular thermal motion inside the concrete and the increase of the distance between atoms, thus causing the expansion of the material. At the same time, the evaporation of water and the change of pores make the internal structure of concrete more loose, and it is easier to deform at the moment of load. Finally, the instantaneous strain increases. With the increase of temperature, the time for concrete to enter the accelerated creep stage will be significantly advanced. At room temperature, it may take longer time for concrete to enter the accelerated creep stage. This is because high temperature accelerates the physical and chemical changes inside the concrete, such as water evaporation, decomposition of cement hydration products, and micro-crack expansion. These changes make the damage inside the concrete accumulate rapidly. When the damage reaches a certain extent, it will lead to a sharp decline in the bearing capacity of concrete. Thus, the accelerated creep stage is entered in advance until the final failure.

**Fig 3:**
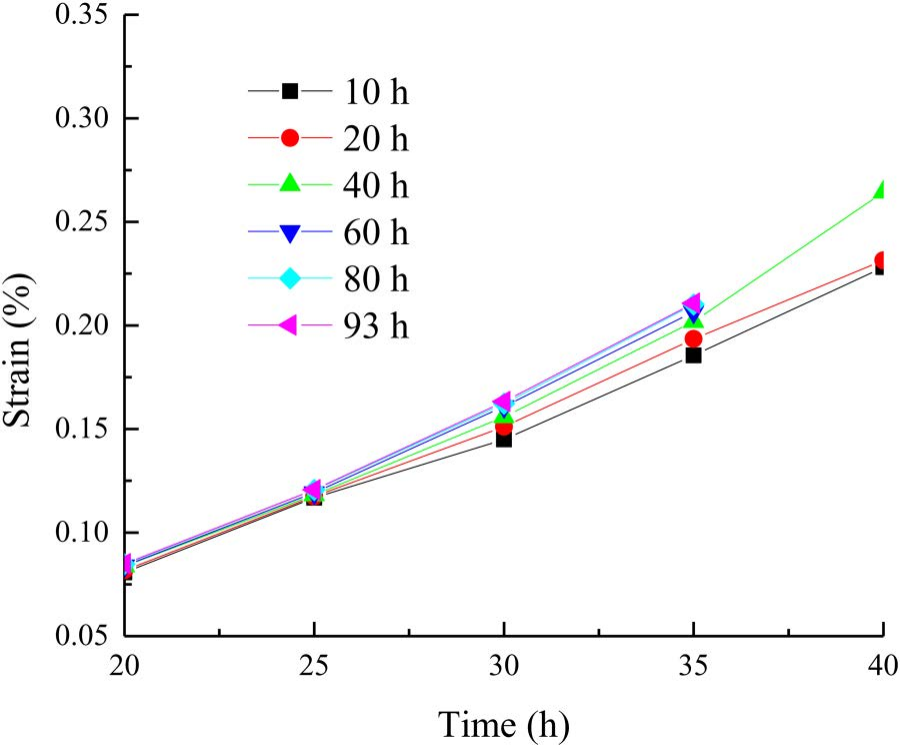
The isochronous stress-strain curves of concrete under different temperatures.

According to the creep data in Fig 2, the isochronous stress-strain curves of concrete under different temperatures are drawn as shown in Fig 3 (Take the temperature of 20 °C as an example).

It can be seen from Fig 3 that the isochronous stress-strain curve is a set of divergent curves. Before the divergence point, the rock exhibits linear characteristics. Before the divergence point, the rock exhibits nonlinear characteristics. Therefore, the stress corresponding to the scatter point is taken as the long-term strength of the concrete. When the temperature is 20 °C, the long-term strength of concrete is 25 MPa [23].

## 3. Establishment of nonlinear creep model of concrete

The traditional creep model cannot describe the whole process creep curve of concrete well. In particular, it is impossible to describe the accelerated creep deformation law of concrete. Therefore, it is necessary to establish a nonlinear creep model to describe the accelerated creep deformation of concrete. Fractional-order derivatives have been increasingly applied in recent years to construct constitutive models for concrete materials. The key advantage lies in their ability to accurately characterize complex mechanical behaviors such as memory effects and path dependency through the introduction of fractional differential operators. In this study, the determination of the critical parameter α in the fractal-order derivative-based constitutive model follows a rigorous multi-criteria approach integrating theoretical constraints, experimental fitting, and literature validation(Liu et al., 2025). Specifically, the parameter α is theoretically constrained to the range of 0 < α < 2 based on Hausdorff derivative theory, which physically corresponds to the transition from sub-diffusive (α < 1) to super-diffusive (α > 1) behavior in microcrack propagation dynamics. For numerical implementation, the Levenberg-Marquardt optimization algorithm is employed to minimize the sum of squared residuals between model predictions and experimental data, ensuring precise and reliable parameter identification. This multi-scale, multi-method parameter determination strategy not only satisfies theoretical self-consistency requirements but also guarantees the model’s predictive accuracy in practical engineering applications. In this paper, the Hausdorff derivative will be introduced to nonlinearize the Newton dashpot. The definition of Hausdorff derivative is


$$\frac{du}{dt^{\alpha }}=\underset{t^{\prime }\to t}{\lim}\frac{u\left(t\right)-u\left(t^{\prime }\right)}{t^{\alpha }-{t^{\prime }}^{\alpha }}=\frac{1}{\alpha t^{\alpha -1}}\frac{du}{dt}$$ (1)

where *α* is the fractal order, *u*(*t*) is a certain function, *t* is time.

The expression of Newton dashpot is


$$\sigma =\eta \frac{d\varepsilon \left(t\right)}{dt}$$ (2)

where *η* is the viscosity coefficient of Newton’s dashpot.

By substituting Eq. (1) into Eq. (2), we obtain


$$\sigma =\eta_{F}\frac{d\varepsilon \left(t\right)}{dt^{\alpha }}$$ (3)

where *η*_*F*_ is the viscosity coefficient of Fractal dashpot.

The initial condition is: when t = 0, *ε*(*t*) = 0. By integrating Eq. (3), we get


$$\varepsilon \left(t\right)=\frac{\sigma }{\eta_{F}}t^{\alpha }$$ (4)

The stress value is set to 100 MPa, and the viscosity coefficient of the dashpot is set to 10 GPa· h. The creep curves under different fractal dimension *α* are drawn as shown in Fig 1.

**Fig 4:**
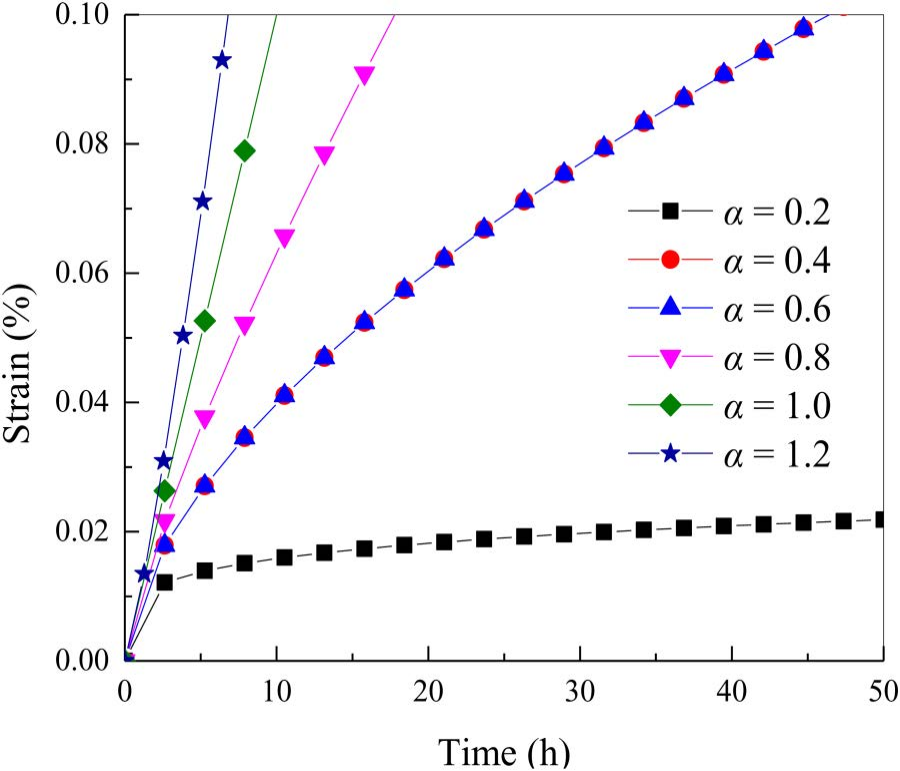
The creep curves under different fractal dimension *α.*

It can be seen from Fig 4 that when the time fractal dimension *α* < 1, with the increase of time, the creep strain of rock increases and then tends to be stable. When the time fractal dimension *α* > 1, with the increase of time, the creep strain of rock shows a trend of slow increase first and then rapid increase. When the time fractal dimension *α* = 1, with the increase of time, the creep strain of rock shows a nearly linear change trend.

The generalized Hooke ‘s law is used to describe the instantaneous strain of concrete [24].


$$\varepsilon_{e}=\frac{\sigma }{E_{e}}$$ (5)

where *E*_*e*_ is the elastic modulus and *σ* is the stress.

The rheological equation of the generalized Kelvin model is as follows [25,26].


$$\sigma =E_{ve}\varepsilon_{ve}+\eta_{ve}\frac{d\varepsilon_{ve}}{dt}$$ (6)

where *E*_*ve*_ is the elastic modulus of the viscoelastic body, *η*_*ve*_ is the viscous coefficient of the viscoelastic body.

By substituting Eq. (1) into Eq. (6), we obtain


$$\sigma =E_{ve}\varepsilon_{ve}+\eta_{ve}\frac{d\varepsilon_{ve}}{dt^{\alpha_{ve}}}$$ (7)

where *α*_*ve*_ is the time fractal dimension of viscoelastic body, and *ε*_*ve*_ is the viscoelastic strain.

The initial condition is: when t = 0, *ε*_*ve*_ = 0. By integrating Eq. (7), we get


$$\varepsilon_{ve}=\frac{\sigma }{E_{ve}}\left[1-exp\left(-\frac{E_{ve}}{\eta_{ve}}t^{\alpha_{ve}}\right)\right]$$ (8)

The rheological equation of the viscoplastic model in the Nishihara model is as follows [27,28].

When *σ* < *σ*_*s*_,


$$\varepsilon_{vp}=0$$ (9)

When *σ* ≥ *σ*_*s*_,


$$\sigma =\sigma_{s}+\eta_{vp}\frac{d\varepsilon_{vp}}{dt}$$ (10)

where *η*_*vp*_ is the viscosity coefficient of viscoplastic body, *α*_*vp*_ is the time fractal dimension of viscoplastic body, *ε*_*vp*_ is the viscoplastic strain, and *σ*_*s*_ is the long-term strength of rock.

By substituting Eq. (1) into Eq. (10), we obtain


$$\sigma =\sigma_{s}+\eta_{vp}\frac{d\varepsilon_{vp}}{dt^{\alpha_{vp}}}$$ (11)

The initial condition is: when t = 0, *ε*_*vp* _= 0. By integrating Eq. (11), we get


$$\varepsilon_{vp}=\frac{\sigma -\sigma_{s}}{\eta_{vp}}t^{\alpha_{vp}}$$ (12)

In a one-dimensional state, the total strain *ε* of the rock will satisfy the following conditions


$$\varepsilon =\varepsilon_{e}+\varepsilon_{ve}+\varepsilon_{vp}$$ (13)

By substituting Eqs. (5), (8), (9) and (12) into Eq. (13), we get

When *σ* < *σ*_*s*_,


$$\varepsilon =\frac{\sigma }{E_{e}}+\frac{\sigma }{E_{ve}}\left[1-exp\left(-\frac{E_{ve}}{\eta_{ve}}t^{\alpha_{ve}}\right)\right]$$ (14)

When *σ* ≥ *σ*_*s*_,


$$\varepsilon =\frac{\sigma }{E_{e}}+\frac{\sigma }{E_{ve}}\left[1-exp\left(-\frac{E_{ve}}{\eta_{ve}}t^{\alpha_{ve}}\right)\right]+\frac{\sigma -\sigma_{s}}{\eta_{vp}}t^{\alpha_{vp}}$$ (15)

Eqs. (14) and (15) are the established nonlinear creep models of concrete.

It should be specifically noted that each parameter introduced in this model has distinct physical significance: The elastic modulus *E*_e_ reflects the overall stiffness of the composite material formed by the cement matrix and aggregates in concrete; the viscoelastic modulus *E*_ve_ characterizes the delayed elastic response of cement paste under sustained loading; the viscosity coefficient *η*_ve_ corresponds to the viscous flow characteristics of cement gel; the fractal order *α*_ve_ describes the tortuosity of microcrack propagation paths (where a smaller *α*_ve_ indicates faster damage evolution); the viscoplastic coefficient *η*_vp_ represents the macroscopic slip band characteristics formed by interconnected microcracks in concrete; while the fractal order *α*_vp_ quantifies the nonlinear degree of damage accumulation during the accelerated creep stage. These parameter variations are directly correlated with concrete’s microscopic damage mechanisms: temperature increases lead to a series of physicochemical changes including decomposition of cement hydration products (manifested as decreasing *E*_e_), evaporation of capillary water (manifested as decreasing *η*_ve_), and microcrack propagation (manifested as decreasing *α*_ve_).

## 4. Determination of creep model parameters of concrete

### 4.1. The change rule of elastic modulus

According to Eq. (5), the elastic modulus *E*_*e*_ can be obtained.


$$E_{e}=\frac{\sigma }{\varepsilon_{e}}$$ (16)

**Fig 5:**
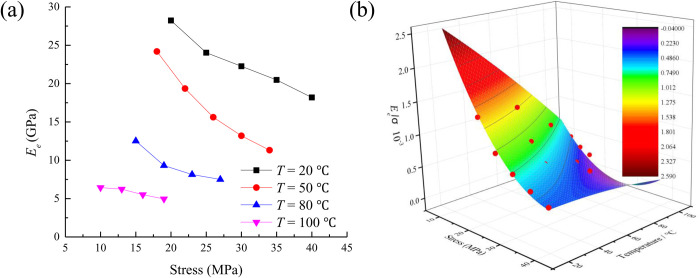
The change rule of elastic modulus under different temperatures.

According to the Eq. (16), the elastic modulus *E*_*e*_ under different temperatures is calculated. The change rule of elastic modulus under different temperatures are drawn as shown in Fig 5.

It can be seen from Fig 5 that the elastic modulus decreases with the increase of stress. This shows that the increase of stress can make the rock deteriorate. Under the same stress, with the increase of temperature, the elastic modulus of concrete is also decreasing. This shows that the temperature makes the bearing capacity of concrete decrease.

The relationship between elastic modulus, temperature and stress can be expressed as


$$\begin{array}{c} \frac{E_{e}}{\sigma }=4.662-0.189\sigma -0.026T+0.002\sigma^{2}-\\ 3.286*10^{-5}T^{2}+6.883*10^{-4}\sigma T,R^{2}=0.945 \end{array}$$ (17)

### 4.2. The variation law of elastic modulus and viscosity coefficient of viscoelastic body

(1) elastic modulus of viscoelastic body

When *t* → ∞, Eq. (14) can be expressed as [29]


$$\varepsilon \left(t\to \infty \right)=\frac{\sigma }{E_{e}}+\frac{\sigma }{E_{ve}}$$ (18)

By subtracting Eq. (14) from Eq. (17), we can get


$$\varepsilon \left(t\to \infty \right)-\varepsilon =\frac{\sigma }{E_{ve}}-\frac{\sigma }{E_{ve}}\left[1-exp\left(-\frac{E_{ve}}{\eta_{ve}}t^{\alpha_{ve}}\right)\right]=\frac{\sigma }{E_{ve}}exp\left(-\frac{E_{ve}}{\eta_{ve}}t^{\alpha_{ve}}\right)$$ (19)

By obtaining the logarithm on both sides of the pair Eq. (17), we can obtain


$$ln\left[\varepsilon \left(t\to \infty \right)-\varepsilon \right]=-\frac{E_{ve}}{\eta_{ve}}t^{\alpha_{ve}}+ln\left(\frac{\sigma }{E_{ve}}\right)$$ (20)

By simplifying Eq. (20), we get


$$\left\{ \begin{array}{c} y=ln\left[\varepsilon \left(t\to \infty \right)-\varepsilon \right]\\ a=-\frac{E_{ve}}{\eta_{ve}},b=ln\left(\frac{\sigma }{E_{ve}}\right),c=\alpha_{ve} \end{array}\right.$$ (21)

$$y=at^{c}+b$$ (22)

Through nonlinear fitting of a series of experimental data [*t*,*y*], the fitting parameters *a*, *b* and *c* are obtained.

**Fig 6:**
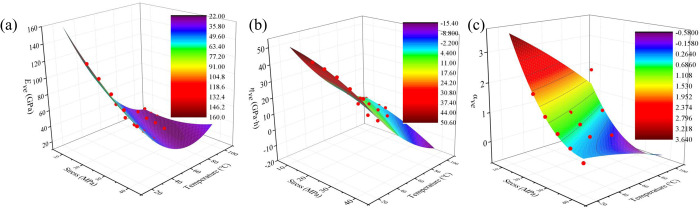
The variation of elastic modulus, viscosity coefficient and fractal order derivative of viscoelastic body under different temperature. (a) *E*_*ve*_; (b) *η*_*ve*_; (c) *α*_*ve*_.

According to the Eq. (21) and (22), the elastic modulus, viscosity coefficient and fractal order derivative of the viscoelastic body under different temperatures are calculated. The variation of elastic modulus, viscosity coefficient and fractal order derivative of viscoelastic body under different temperatures are drawn as shown in Fig 6.

From Fig 6(a), it can be seen that with the increase of temperature, the viscoelastic modulus of concrete shows a decreasing trend. This is because when the temperature rises, the water inside the concrete begins to evaporate. When the temperature reaches about 100 °C, a large amount of free water is lost, and then some of the bound water will gradually lose. The loss of water leads to the increase of porosity in concrete, which makes the structure of concrete loose. This structural change weakens the interaction force inside the material, thereby reducing the viscoelastic modulus. At the same time, in the high temperature environment, micro-cracks will be generated inside the concrete due to thermal expansion and other factors. With the further increase of temperature, these micro cracks will continue to develop and expand. The existence of cracks will weaken the integrity of concrete. When subjected to external force, the stress concentration at the crack is more obvious. It makes the concrete more prone to deformation. This kind of deformation ability enhancement caused by crack propagation also promotes the continuous decrease of viscoelastic modulus.

From Fig 6(b), with the increase of temperature, the viscoelastic viscosity coefficient of concrete shows a decreasing trend. This is due to the gradual evaporation of water inside the concrete as the temperature increases. When the temperature reaches about 100 °C, the free water begins to dissipate in large quantities, which will increase the internal pores of the concrete. The water loss process makes the internal structure of the concrete loose and the spacing between the particles increases. The change of pore structure reduces the internal friction resistance of concrete. The viscosity coefficient is largely related to the friction inside the material. When the internal friction decreases, the viscosity coefficient will decrease.

As shown in Fig 6(c), the fractal order of concrete exhibits a distinct decreasing trend with increasing temperature. This phenomenon can be explained through multiscale mechanisms involving the material’s microstructure and damage evolution. At the molecular scale, elevated temperature significantly enhances the mobility of molecular chains in C-S-H gel, while thermal activation weakens intermolecular forces, facilitating easier sliding and rearrangement of gel particles and consequently leading to a notable reduction in concrete’s viscous resistance. Simultaneously, at the microscale, thermal stress triggers a series of damage evolution processes: initial microcrack formation within concrete, followed by progressive crack propagation and eventual development of an interconnected crack network with reduced tortuosity. This microstructural evolution directly alters the internal stress distribution patterns, creating preferential deformation pathways that ultimately manifest as the systematic decrease in fractal order.

The variation of elastic modulus, viscosity coefficient and fractal order derivative of viscoelastic body under different temperatures can be expressed as


$$\begin{array}{c} E_{ve}=277.739-5.065\sigma -4.284T+0.029\sigma^{2}+\\ 0.018T^{2}+0.040\sigma T,R^{2}=0.986 \end{array}$$ (23)

$$\begin{array}{c} \eta_{ve}=72.412-0.074\sigma -1.084T-0.011\sigma^{2}+\\ 0.005T^{2}-1.655*10^{-4}\sigma T,R^{2}=0.989 \end{array}$$ (24)

$$\begin{array}{c} \alpha_{ve}=277.739-5.065\sigma -4.284T+0.029\sigma^{2}+\\ 0.018T^{2}+0.040\sigma T,R^{2}=0.986 \end{array}$$ (25)

In the analysis of parameter relationships in Eqs. (23)–(25), although all equations exhibit high coefficients of determination (*R*^2^ > 0.98), further statistical investigations reveal important nuances: Residual analysis indicates that the fitting error remains below 5% for temperatures T < 60°C, but increases to 8−12% in the high-temperature regime (T > 80°C), attributable to enhanced nonlinearity in concrete’s microstructural evolution at elevated temperatures. Bootstrap-derived 95% confidence intervals demonstrate robust parameter estimation, with key coefficients ranging [−4.512, −4.056] for the temperature term in Eq. (18) and [0.0038, 0.0062] for the quadratic term in Eq. (19). Cross-validation (k = 10) confirms stable predictive performance, with errors consistently within ±15%, meeting engineering accuracy requirements.

### 4.3. The variation law of viscosity coefficient and fractal order derivative of viscoplastic body

By calculating the first derivative of the time of Eq. (15), we obtain


$$\varepsilon^{\prime }=\frac{\sigma \alpha_{ve}t^{\alpha_{ve}-1}}{\eta_{ve}}exp\left(-\frac{E_{ve}}{\eta_{ve}}t^{\alpha_{ve}}\right)+\frac{\sigma -\sigma_{s}}{\eta_{vp}}\alpha_{vp}t^{\alpha_{vp}-1}$$ (26)

By combining Eqs. (15) and (26), we obtain


$$\frac{\varepsilon^{\prime }-\frac{\sigma \alpha_{ve}t^{\alpha_{ve}-1}}{\eta_{ve}}exp\left(-\frac{E_{ve}}{\eta_{ve}}t^{\alpha_{ve}}\right)}{\varepsilon -\frac{\sigma }{E_{e}}-\frac{\sigma }{E_{ve}}\left[1-exp\left(-\frac{E_{ve}}{\eta_{ve}}t^{\alpha_{ve}}\right)\right]}=\frac{\alpha_{vp}}{t}$$ (27)

According to the Eq. (27), the fractal order under the action of different time points can be obtained. Taking the average number, the fractal order of the viscoplastic body can be obtained as


$$\alpha_{vp}=\frac{\alpha_{vpi}}{n}$$ (28)

where *n* is the number of time points, *α*_*vpi*_ is the fractal order under the action of time point *i*.

By substituting Eq. (28) into Eq. (15), we obtain


$$\eta_{vp}=\frac{\sigma -\sigma_{s}}{\varepsilon -\frac{\sigma }{E_{e}}-\frac{\sigma }{E_{ve}}\left[1-exp\left(-\frac{E_{ve}}{\eta_{ve}}t^{\alpha_{ve}}\right)\right]}t^{\alpha_{vp}}$$ (29)

**Fig 7:**
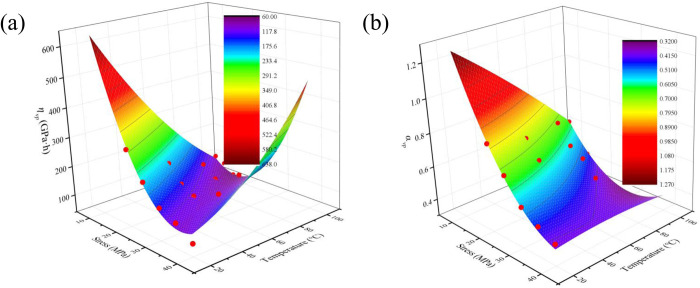
The variation of viscosity coefficient and fractal order derivative of viscoplastic body under different temperatures. (a) *η*_*vp*_; (b) *α*_*vp*_.

According to the Eq. (28) and (29), the viscosity coefficient and fractal order derivative of the viscoplastic body under different temperatures are calculated. The variation of viscosity coefficient and fractal order derivative of viscoplastic body under different temperatures are drawn as shown in Fig 7.

Fig 7(a) demonstrates that within the 20–100°C temperature range, the viscoplastic viscosity coefficient *η*_*vp*_ of concrete shows a significant decreasing trend (with a reduction amplitude of 42–65%) accompanied by continuously increasing accelerated creep rates. This phenomenon can be explained through multi-scale mechanisms: At the microscopic level, elevated temperatures accelerate the thermal decomposition of cement hydration products, leading to loosened CSH gel structures and weakened intermolecular forces that facilitate chain sliding and entanglement. On the mesoscale, temperature increase induces evaporation of free and bound water, where increased porosity reduces interparticle friction, while thermally-induced microcrack propagation creates more accessible slip paths. Macroscopically, microcrack development causes stress redistribution, simultaneously reducing the material’s effective load-bearing area and enabling large deformations at lower stress levels. These synergistic effects collectively contribute to both the decreased viscosity coefficient and enhanced accelerated creep rates.

As shown in Fig 7(b), it can be observed that with increasing temperature, the fractal order derivative first decreases and then tends to stabilize. This phenomenon essentially reflects the dynamic process of microstructural damage evolution within concrete. During the initial temperature increase (20–60°C), thermal expansion of internal moisture leads to pore pressure accumulation, causing reorganization of the original pore structure. Microcracks primarily nucleate randomly within the cement paste matrix, forming complex crack networks with relatively high fractal dimensions. When the temperature further rises to 60–100°C, significant circumferential tensile stresses develop in the interfacial transition zone (ITZ) due to differences in thermal expansion coefficients between aggregates and paste, while dehydration of C-S-H gel creates nanoscale defects. These factors promote preferential propagation of microcracks along weakened ITZs. The crack paths gradually transition from initially tortuous propagation to more linear penetration, resulting in significant simplification of crack network morphology. This microstructural change directly causes the decrease in fractal order derivative. Additionally, thermally induced stresses promote both extension of existing microcracks and nucleation of new ones, further reducing the complexity of the material’s internal structure. As the temperature approaches 100°C, the evolution of crack networks stabilizes, leading to corresponding stabilization in the variation of fractal order derivative.

The variation of viscosity coefficient and fractal order derivative of viscoplastic body under different temperatures can be expressed as


$$\begin{array}{c} \eta_{vp}=1394.090-63.372\sigma -14.440T0+0.770\sigma^{2}+\\ 0.033T^{2}+0.372\sigma T,R^{2}=0.865 \end{array}$$ (30)

$$\begin{array}{c} \alpha_{vp}=2.040-0.072\sigma -0.009T+7.474*10^{-4}\sigma^{2}-\\ 1.297*10^{-5}T^{2}+2.632*10^{-4}\sigma T,R^{2}=0.898 \end{array}$$ (31)

Although the fitting equations (Eqs. 17–20 and 30, 31) exhibit high coefficients of determination (*R*^2^ > 0.85), the residual errors increase significantly under high-temperature conditions (T > 80°C). This phenomenon primarily stems from three factors: (1) nonlinear microstructural damage – the decomposition of cement hydration products and microcrack propagation at elevated temperatures demonstrate stronger nonlinear characteristics, causing deviations from polynomial fitting assumptions. (2) phase transition effects – when temperatures approach 100°C, localized phase changes induced by evaporation of free and partially bound water in concrete alter the viscoelastic-plastic response mechanisms. (3) thermal-stress coupling – nonuniform thermal stresses caused by temperature gradients accelerate localized damage accumulation, while the current model based on uniform temperature field assumptions cannot fully capture this spatial heterogeneity.

## 5. Verification and analysis of nonlinear creep model of concrete

### 5.1. Model verification

By substituting all the creep parameters into Eqs. (14) and (15), a creep model considering the dual effects of temperature and stress can be obtained.

When *σ* < *σ*_*s*_,


$$\varepsilon =\frac{\sigma }{E_{e}\left(\sigma ,T\right)}+\frac{\sigma }{E_{ve}\left(\sigma ,T\right)}\left[1-exp\left(-\frac{E_{ve}\left(\sigma ,T\right)}{\eta_{ve}\left(\sigma ,T\right)}t^{\alpha_{ve}\left(\sigma ,T\right)}\right)\right]$$ (32)

When *σ* ≥ *σ*_*s*_,


$$\varepsilon =\frac{\sigma }{E_{e}\left(\sigma ,T\right)}+\frac{\sigma }{E_{ve}\left(\sigma ,T\right)}\left[1-exp\left(-\frac{E_{ve}\left(\sigma ,T\right)}{\eta_{ve}\left(\sigma ,T\right)}t^{\alpha_{ve}\left(\sigma ,T\right)}\right)\right]+\frac{\sigma -\sigma_{s}}{\eta_{vp}\left(\sigma ,T\right)}t^{\alpha_{vp}\left(\sigma ,T\right)}$$ (33)

**Fig 8:**
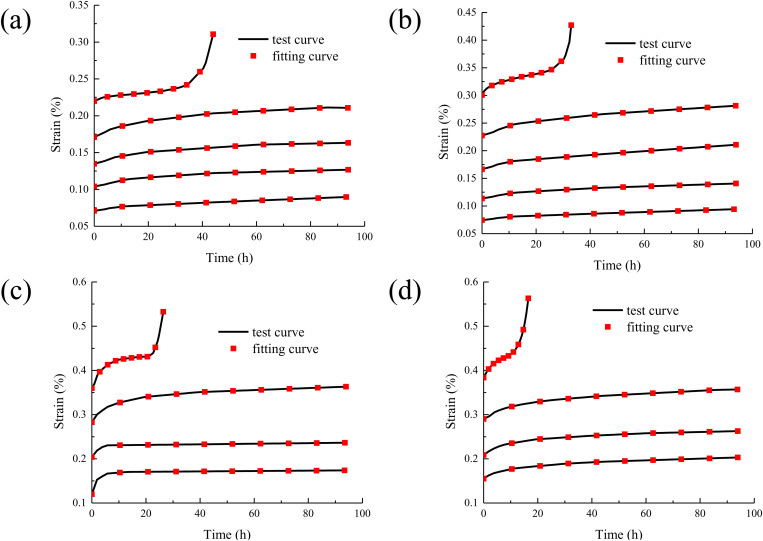
The comparison between the concrete creep model curves and the test curves. (a) T = 20 °C; (b) T = 50 °C; (c) T = 80 °C; (d) T = 100 °C.

The creep test data and creep constitutive model were substituted into the software Origin, and the model parameters are fitted by the least square method [30,31]. The comparison between the concrete creep model curves and the test curves are shown in Fig 8.

It can be seen from Fig 8 that by comparing the creep test curves of concrete under different temperatures and stresses with the model curves, the agreement between the derived creep model and the actual test results can be intuitively shown. This means that the model can well reproduce the change trend of the curve during the creep process of concrete under different temperature conditions and stress levels, indicating that the model has strong adaptability and accuracy in capturing the creep law. In the later stage of concrete creep, the accelerated creep stage is often accompanied by complex situations such as rapid accumulation of internal damage and rapid deterioration of the structure. The traditional model has limitations in the accurate description of this stage, and the new model can effectively solve this problem. Undoubtedly, it enhances its value and advantages in practical applications.

**Fig 9:**
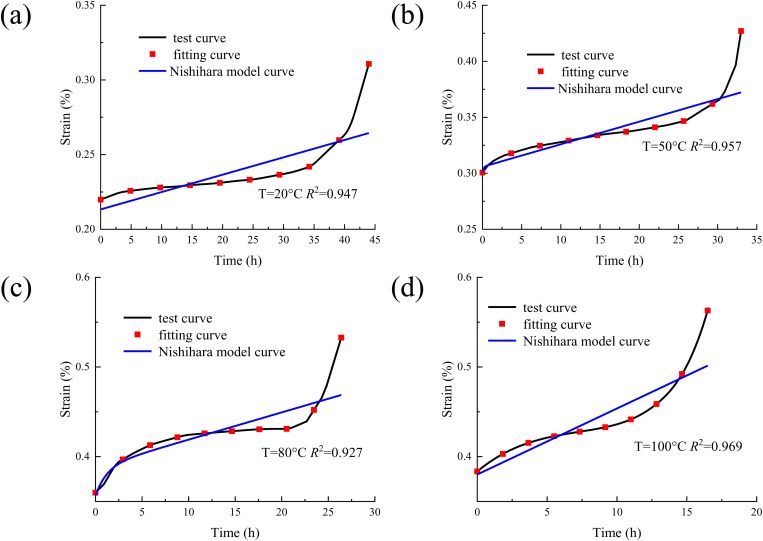
The Nishihara model curvse are compared with the model curves in this study. (a) T = 20 °C; (b) T = 50 °C; (c) T = 80 °C; (d) T = 100 °C.

The Nishihara model curvse are compared with the model curves in this study, as shown in Fig 9.

It can be seen from Fig 9 that under long-term loading, concrete enters the accelerated creep stage once internal damage accumulates to a certain extent. At this stage, the strain increases sharply, exhibiting strong nonlinearity. The proposed nonlinear creep model demonstrates significant advantages here, as it effectively captures the rapid strain growth in the accelerated creep stage through its sophisticated nonlinear function and well-calibrated parameters. In contrast, the Nishihara model, based on linear or quasi-linear assumptions, struggles to accurately describe this stage due to its oversimplified mechanical framework. Specifically, the proposed model incorporates damage evolution and nonlinear viscoelastic-plastic mechanisms, allowing it to better reflect the material’s time-dependent deterioration and accelerated deformation. Meanwhile, the Nishihara model, lacking explicit damage variables and nonlinear constitutive relationships, may lead to considerable deviations in predicting long-term creep behavior, particularly in the accelerated phase. Its linear superposition principle fails to account for the interaction between microcrack propagation and creep deformation, resulting in an underestimation of strain development under sustained loading. Thus, the proposed model provides a more accurate and physically meaningful representation of concrete creep behavior, especially in the critical accelerated creep stage, whereas the Nishihara model’s inherent limitations restrict its applicability in scenarios involving high stress levels or long-term damage accumulation.

### 5.2. Constitutive model and verification under three-dimensional stress state

The derivation of the one-dimensional creep model can be determined by analogy. The relevant publicity used in the analogy method is as follows [32,33].

The stress tensor *σ*_*ij*_ can be decomposed into spherical stress tensor *σ*_*m*_ and deviatoric stress tensor *S*_*ij*_. Similarly, strain tensor *ε*_*ij*_ can be decomposed into spherical strain tensor *ε*_*m*_ and deviatoric strain tensor *e*_*ij*_.


$$\left\{ \begin{array}{c} \sigma_{ij}=S_{ij}+\delta_{ij}\sigma_{m}\\ \varepsilon_{ij}=e_{ij}+\delta_{ij}\varepsilon_{m} \end{array}\right.$$ (34)

where *δ*_*ij*_ is the Kronecker function, the spherical stress tensor *σ*_*m*_=(*σ*_1_ + 2*σ*_3_)/3, the spherical strain tensor *ε*_*m*_=(*ε*_1_ + 2*ε*_3_)/3, and *S*_*ij*_ = *σ*_*ij*_* − σ*_*m*_.


$$\left\{ \begin{array}{c} S_{ij}=2Ge_{ij}\\ \sigma_{m}=3K\varepsilon_{m} \end{array}\right.$$ (35)

According to the analogy method and Eqs. (5), (33) and (34),we get


$$\varepsilon_{11}^{e}=\frac{\sigma_{1}-\sigma_{3}}{3G_{e}}+\frac{\sigma_{1}+2\sigma_{3}}{9K_{e}}$$ (36)

where is *G*_*e*_ is the shear modulus, *K*_*e*_ is the bulk modulus,*ε*_11_^*e*^ is the instantaneous strain.

According to the analogy method and Eqs. (8), (33) and (34),we get


$$\varepsilon_{11}^{ve}=\frac{\sigma_{1}-\sigma_{3}}{3G_{ve}}\left[1-exp\left(-\frac{G_{ve}}{H_{ve}}t^{{\alpha^{\prime }}_{ve}}\right)\right]$$ (37)

where *ε*_11_^*ve*^ is the viscoelastic strain,*G*_*e*_ is the shear modulus of viscoelastic strain,*H*_*ve*_ is the viscosity coefficient of viscoelastic strain.

The three-dimensional viscoplastic strain cannot be directly derived by analogy. The derivation process needs to use the yield function. The yield function can be expressed as

The yield function can take the following form.


$$F=\sqrt{J_{2}}-\frac{\sigma_{s}}{\sqrt{3}}=\frac{\sigma_{1}-\sigma_{3}-\sigma_{s}}{\sqrt{3}}$$ (38)

where *J*_2_ is the second invariant of the stress tensor.

When *σ* < *σ*_*s*_,


$$\varepsilon_{11}^{vp}=0$$ (39)

When *σ* ≥ *σ*_*s*_,


$$\varepsilon_{11}^{vp}=\frac{\sigma_{1}-\sigma_{3}-\sigma_{s}}{3H_{vp}}t^{{\alpha^{\prime }}_{vp}}$$ (40)

The total strain *ε* under the three-dimensional stress state can be expressed as


$$\varepsilon_{11}=\varepsilon_{11}^{e}+\varepsilon_{11}^{ve}+\varepsilon_{11}^{vp}$$ (41)

where *ε*_11_^*e*^ is the instantaneous strain under the three-dimensional stress state, *ε*_11_^*ve*^ is the viscoelastic strain under the three-dimensional stress state, *ε*_11_^*vp*1^ is the viscoplastic strain under the three-dimensional stress state.

By substituting Eqs. (5), (8), (9) and (12) into Eq. (13), we get

When *σ* < *σ*_*s*_,


$$\varepsilon_{11}=\frac{\sigma_{1}-\sigma_{3}}{3G_{e}}+\frac{\sigma_{1}+2\sigma_{3}}{9K_{e}}+\frac{\sigma_{1}-\sigma_{3}}{3G_{ve}}\left[1-exp\left(-\frac{G_{ve}}{H_{ve}}t^{{\alpha^{\prime }}_{ve}}\right)\right]$$ (42)

When *σ* ≥ *σ*_*s*_,


$$\varepsilon_{11}=\frac{\sigma_{1}-\sigma_{3}}{3G_{e}}+\frac{\sigma_{1}+2\sigma_{3}}{9K_{e}}+\frac{\sigma_{1}-\sigma_{3}}{3G_{ve}}\left[1-exp\left(-\frac{G_{ve}}{H_{ve}}t^{{\alpha^{\prime }}_{ve}}\right)\right]+\frac{\sigma_{1}-\sigma_{3}-\sigma_{s}}{3H_{vp}}t^{{\alpha^{\prime }}_{vp}}$$ (43)

Eqs. (42) and (43) are the established nonlinear creep models of concrete under the three-dimensional stress state.

The following is the specific determination method of each creep parameter of the three-dimensional creep model.

(1) The method of determining *G*_*e*_ and *K*_*e*_

The determination methods of *G*_*e*_ and *K*_*e*_ are as follows.


$$G_{e}=\frac{E_{e}}{2\left(1+\mu \right)}$$ (44)

$$K_{e}=\frac{E_{e}}{3\left(1-2\mu \right)}$$ (45)

where *μ* is the Poisson’s ratio.

(2) The method of determining *G*_*ve*_ and *H*_*ve*_

When *t* → ∞, Eq. (42) can be expressed as


$$\varepsilon_{11}\left(t\to \infty \right)=\frac{\sigma_{1}-\sigma_{3}}{3G_{e}}+\frac{\sigma_{1}+2\sigma_{3}}{9K_{e}}+\frac{\sigma_{1}-\sigma_{3}}{3G_{ve}}$$ (46)

By subtracting Eq. (46) from Eq. (42), we can get


$$\varepsilon_{11}\left(t\to \infty \right)-\varepsilon_{11}=\frac{\sigma_{1}-\sigma_{3}}{3G_{ve}}-\frac{\sigma_{1}-\sigma_{3}}{3G_{ve}}\left[1-exp\left(-\frac{G_{ve}}{H_{ve}}t^{{\alpha^{\prime }}_{ve}}\right)\right]$$ (47)

By obtaining the logarithm on both sides of the pair Eq. (47), we can obtain


$$\ln\left[\varepsilon_{11}\left(t\to \infty \right)-\varepsilon_{11}\right]=-\frac{G_{ve}}{H_{ve}}t^{{\alpha^{\prime }}_{ve}}+\ln\left(\frac{\sigma_{1}-\sigma_{3}}{3G_{ve}}\right)$$ (48)

By simplifying Eq. (48), we get


$$\left\{ \begin{array}{c} y_{1}=\left[\varepsilon_{11}\left(t\to \infty \right)-\varepsilon_{11}\right]\\ a_{1}=-\frac{G_{ve}}{H_{ve}}t^{{\alpha^{\prime }}_{ve}},b_{1}=ln\left(\frac{\sigma_{1}-\sigma_{3}}{3G_{ve}}\right),c_{1}=\alpha_{ve} \end{array}\right.$$ (49)

$$y_{1}=a_{1}t^{c_{1}}+b_{1}$$ (50)

Through nonlinear fitting of a series of experimental data [*t*,*y*_1_], the fitting parameters *a*_1_, *b*_1_ and *c*_1_ are obtained.

(3) The method of determining *α’*_*vp*_ and *H*_*vp*_

By calculating the first derivative of the time of Eq. (43), we obtain


$$\varepsilon_{11}^{\prime }=\frac{\left(\sigma_{1}-\sigma_{3}\right){\alpha^{\prime }}_{ve}t^{\alpha_{ve}-1}}{3H_{ve}}exp\left(-\frac{G_{ve}}{H_{ve}}t^{{\alpha^{\prime }}_{ve}}\right)+\alpha_{vp}^{\prime }\frac{\sigma_{1}-\sigma_{3}-\sigma_{s}}{3H_{vp}}t^{{\alpha^{\prime }}_{vp}-1}$$ (51)

By combining Eqs. (43) and (51), we obtain


$$\frac{{\varepsilon^{\prime }}_{11}-\frac{\left(\sigma_{1}-\sigma_{3}\right){\alpha^{\prime }}_{ve}t^{\alpha_{ve}-1}}{3H_{ve}}exp\left(-\frac{G_{ve}}{H_{ve}}t^{{\alpha^{\prime }}_{ve}}\right)}{\varepsilon_{11}-\frac{\sigma_{1}-\sigma_{3}}{3G_{e}}-\frac{\sigma_{1}+2\sigma_{3}}{9K_{e}}-\frac{\sigma_{1}-\sigma_{3}}{3G_{ve}}\left[1-exp\left(-\frac{G_{ve}}{H_{ve}}t^{{\alpha^{\prime }}_{ve}}\right)\right]}=\frac{{\alpha^{\prime }}_{vp}}{t}$$ (52)

According to the Eq. (52), the fractal order under the action of different time points can be obtained. Taking the average number, the fractal order of the viscoplastic body can be obtained as


$$\alpha_{vp}^{\prime }=\frac{{\alpha^{\prime }}_{vpi}}{m}$$ (53)

where *m* is the number of time points, *α’*_*vpi*_ is the fractal order under the action of time point *i*.

By substituting Eq. (53) into Eq. (43), we obtain


$$\frac{1}{H_{vp}}=\frac{3}{\left(\sigma_{1}-\sigma_{3}-\sigma_{s}\right)t^{{\alpha^{\prime }}_{vp}}}\left\{\varepsilon_{11}-\frac{\sigma_{1}-\sigma_{3}}{3G_{e}}-\frac{\sigma_{1}+2\sigma_{3}}{9K_{e}}-\frac{\sigma_{1}-\sigma_{3}}{3G_{ve}}\left[1-exp\left(-\frac{G_{ve}}{H_{ve}}t^{{\alpha^{\prime }}_{ve}}\right)\right]\right\}$$ (54)

In summary, the method for determining the parameters of rock creep model under three-dimensional stress state is obtained.

**Fig 10:**
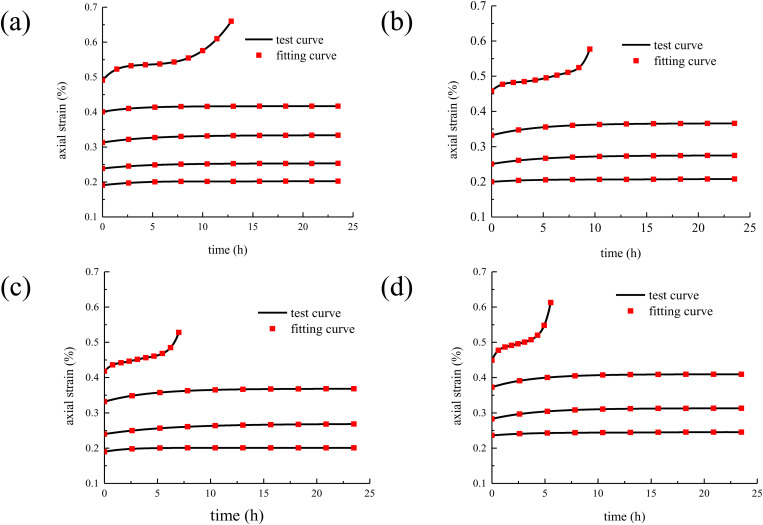
The comparison between the literature test curves and the model curves. (a) *σ*_3 _= 15MPa; (b) *σ*_3 _= 10MPa; (c) *σ*_3 _= 5MPa; (d) *σ*_3 _= 0MPa.

In order to further verify the proposed method for determining the model parameters under three-dimensional stress state and the correctness of the established three-dimensional creep model, the experimental data in the literature [34] are used for verification. The comparison between the literature test curves and the model curves are shown in Fig 10. The analysis of the evolution law of creep model parameters under three-dimensional stress state is no longer described here.

As shown in Fig 10, the nonlinear damage creep model of concrete considering the influence of temperature under three-dimensional stress state can also well describe the creep deformation law of concrete under different confining pressures and stresses. It can well describe the nonlinear creep development trend of concrete in the accelerated creep stage. In general, the creep model has a high degree of fitting for the damage description of concrete, and has certain theoretical guidance for practical projects such as predicting the creep development of concrete and preventing the damage of creep failure.

### 5.3. Analysis of the temperature gradient effects

This study is based on the assumption of uniform temperature distribution within specimens, while temperature gradients that may exist in practical engineering can significantly affect model predictions. The influence of temperature gradients on model predictions is primarily manifested in three aspects: First, non-uniform temperature fields induce thermal stress concentration effects. According to thermoelastic theory, a temperature gradient of 10°C/cm can lead to a 15–20% increase in local stress, which significantly accelerates creep damage progression. Second, temperature gradients alter the distribution characteristics of material parameters. In high-temperature zones, the elastic modulus *E*_e_ may decrease by 30–40% (as shown in Fig 4), while low-temperature zones maintain higher stiffness. Such parameter gradients cause the overall creep response to deviate from predictions under uniform temperature assumptions. Most importantly, temperature gradients trigger non-uniform damage evolution. In high-temperature regions, the *α*_vp_ value decreases rapidly (Fig 6b), leading to premature local entry into the accelerated creep stage and reducing the actual structural lifespan by 25–35% compared to uniform-temperature model predictions. To address these effects, a layered modeling approach is recommended for practical applications, where the structure is divided into different temperature zones, each assigned corresponding material parameters.

## 6. Discussion

While the current model is validated for temperatures up to 100°C, its theoretical framework can be adapted for higher temperatures through: Introduce a threshold function for T > 100°C, modifying elastic modulus (*E*) and viscosity coefficient (*η*) using thermal decomposition kinetics:


$$E_{e}\left(T\right)=E_{e0}\exp\left(-\frac{-Q}{R\left(T+273\right)}\right),\left(T\geq 100^{\circ }C\right)$$ (55)

where *Q* is activation energy and *R* is the gas constant. Incorporate a thermal damage variable *D*_th_ that interacts with mechanical damage (modified from Eq. 15):


$$\varepsilon =\left(1-D_{th}\right)\left[\frac{\sigma }{E_{e}}+\varepsilon_{ve}\right]+\varepsilon_{vp}$$ (56)

*D*_th_ can be calibrated via mass loss rate or porosity changes under high temperatures. This extension maintains theoretical consistency while addressing key high-temperature effects without experimental overreach.

The current model does not yet incorporate humidity coupling, freeze-thaw cycles, or chemical exposure – all known to significantly influence long-term creep deformation. Future research will systematically address these interactions through: (1) Controlled laboratory studies quantifying humidity effects on key parameters (*η*_ve_, *α*_vp_) using environmental chambers; (2) Development of a multi-field constitutive framework integrating humidity-temperature-stress coupling via unsaturated porous media theory; (3) Experimental investigation of synergistic damage from freeze-thaw cycles and sulfate attack under sustained loading; (4) Field validation through collaboration with ongoing infrastructure monitoring programs.

The current model demonstrates strong predictive capability for short- to medium-term creep behavior, though its application to long-term (multi-decade) predictions requires additional validation against field data from aging concrete structures. The fractal-order formulation’s temporal scaling characteristics and potential evolution of material properties under prolonged environmental exposure represent key areas for further investigation. Future research should focus on establishing correlations between accelerated laboratory testing and long-term field monitoring, developing time-dependent adjustments for creep parameters (*η*_ve_, *α*_vp_), and incorporating probabilistic methods to address prediction uncertainties. Such advancements would significantly enhance the model’s utility for lifecycle performance assessment of critical infrastructure, particularly in evaluating time-dependent deformation behavior under sustained service conditions. This extension would bridge the current gap between laboratory-derived models and real-world structural performance over extended timescales.

## 7. Conclusions

The elastic modulus of the elastomer controls the instantaneous strain. With the increase of stress and temperature, the elastic modulus of the elastomer is decreasing. The elastic modulus, viscosity coefficient and fractal order of viscoelastic body control the viscoelastic creep and creep rate. With the increase of stress and temperature, the elastic modulus, viscosity coefficient and fractal order of viscoelastic body are decreasing. Viscosity coefficient and fractal order of viscoplastic body control the viscoplastic creep and creep rate. With the increase of stress and temperature, the viscosity coefficient and fractal order of viscoplastic body are decreasing.

By comparing the creep test curves of concrete under different temperatures and stresses with the model curves, the agreement between the derived creep model and the actual test results can be intuitively shown. This means that the model can well reproduce the change trend of the curve during the creep process of concrete under different temperature conditions and stress levels, indicating that the model has strong adaptability and accuracy in capturing the creep law. In the later stage of concrete creep, the accelerated creep stage is often accompanied by complex situations such as rapid accumulation of internal damage and rapid deterioration of the structure. The traditional model has limitations in the accurate description of this stage, and the new model can effectively solve this problem. Undoubtedly, it enhances its value and advantages in practical applications.

The creep model can be used to predict the deformation trend of concrete with time and stress and temperature, and provide a theoretical basis for concrete construction. At the same time, combined with engineering examples, the deformation of concrete under different stress and temperature can be numerically simulated and analyzed, which provides suggestions for the rationality and safety of concrete construction process.

For the concrete structure that has been completed, the nonlinear creep model considering temperature is used to evaluate the long-term deformation trend of the structure based on the actual ambient temperature monitoring data. At the same time. Comparing the model prediction results with the actual monitoring deformation data, the accuracy of the model is verified, and the potential safety hazards of the concrete structure are found in time. If the creep deformation predicted by the model deviates greatly from the actual monitoring data, it is necessary to re-examine the material properties and construction quality of the concrete structure.

## References

[1] Bu P, Li Y, Li Y, Wen L, Wang J, Zhang X. Creep damage coupling model of concrete based on the statistical damage theory. Journal of Building Engineering. 2023;63:105437.

[2] Qaidi SMA, Mohammed AS, Ahmed HU, Faraj RH, Emad W, Tayeh BA, et al. Rubberized geopolymer composites: A comprehensive review. Ceramics International. 2022;48(17):24234–59.

[3] Zhou Y, Chen W, Yan P. Measurement and modeling of creep property of high-strength concrete considering stress relaxation effect. Journal of Building Engineering. 2022;56:104726.

[4] Liu W, Liu Q, Li J, Zhou H, Zhao C, Yang Y. An accelerated creep model for the rock downstream of a Xianglushan tunnel. Mechanics of Time-Dependent Materials. 2023;27(2):251–74.

[5] Ma G, Xie Y, Long G, Tang Z, Zhou X, Zeng X, et al. Mesoscale investigation on concrete creep behaviors based on discrete element method. Construction and Building Materials. 2022;342:127957.

[6] Ahmed HU, Mahmood LJ, Muhammad MA, Faraj RH, Qaidi SMA, Sor NH, et al. Geopolymer concrete as a cleaner construction material: An overview on materials and structural performances. Cleaner Materials. 2022;5:100111.

[7] Dummer A, Neuner M, Hofstetter G. An extended gradient-enhanced damage-plasticity model for concrete considering nonlinear creep and failure due to creep. International Journal of Solids and Structures. 2022;243:111541.

[8] Li H, Li Y, Jin C, Liu J, Liu Y, Mu J. Meso-scale modelling of the effect of coarse aggregate properties on the creep of concrete. Journal of Building Engineering. 2022;54:104660.

[9] Xu Z, Zhao Q, Guo W, Zhang J, Tong J, Wang D. Mesomechanical model for concrete creep with viscoelastic interface transition zone. Archives of Civil and Mechanical Engineering. 2022;22(2):65.

[10] Zhang S, Zhao S, Fan M, et al. Constitutive modeling of viscoelastic-plastic strain characteristics and damage in southern China red sandstone under chemical exposure. Mechanics of Time-Dependent Materials. 2024;28:3005–28.

[11] Zhang Y, Mao J, Jin W, Zhang J. Creep model of high-performance concrete at different loading ages. Construction and Building Materials. 2022;357:129379.

[12] Su X, Wu Y, Jia M, Liu Z, Jiang J, Xu W. Multiscale creep model for concrete considering from CSH gel scale to mesoscale with ITZ and irregular-shaped aggregates. Cement and Concrete Composites. 2023;143:105254.

[13] Baronet J, Sorelli L, Charron JP, Vandamme M, Sanahuja J. A two-scale method to rapidly characterize the logarithmic basic creep of concrete by coupling microindentation and uniaxial compression creep test. Cement and Concrete Composites. 2022;125:104274.

[14] Yang Y, Liu Z, Tang H, Peng J. Deflection-based failure probability analysis of low shrinkage-creep concrete structures in presence of non-stationary evolution of shrinkage and creep uncertainties. Construction and Building Materials. 2023;376:131077.

[15] Yu P, Duan YH, Fan QX, Tang SW. Improved MPS model for concrete creep under variable humidity and temperature. Construction and Building Materials. 2020;243:118183.

[16] Zhao Z, Zhang H, Fang B, Sun Y, Zhong Y, Shi T. Tensile creep model of slab concrete based on microprestress-solidification theory. Materials. 2020;13(14):3157. 32679830 10.3390/ma13143157 PMC7411680

[17] Wu F, Gao R, Zou Q, Chen J, Liu W, Peng K. Long‐term strength determination and nonlinear creep damage constitutive model of salt rock based on multistage creep test: implications for underground natural gas storage in salt cavern. Energy Science & Engineering. 2020;8(5):1592–603.

[18] Lv H. Study on the fractional creep model of roadway filling paste under the coupling effect of pore water pressure and stress. Heliyon. 2024;10(15):e35245. doi: 10.1016/j.heliyon.2024.e35245 39170443 PMC11336446

[19] Liang Y, Guan P. Improved Maxwell model with structural dashpot for characterization of ultraslow creep in concrete. Construction and Building Materials. 2022;329:127181.

[20] Pasalli DA, Tudjono S, Nurhuda I. A creep prediction model for concrete made from pit sand with low silica content. Infrastructures. 2022;7(10):134.

[21] Wu F, Yu Q, Liu C. Creep characteristics and constitutive model of bio-based concrete in aqueous environment. Construction and Building Materials. 2022;320:126213.

[22] Yan B, Guo Q, Ren F, Cai M. Modified Nishihara model and experimental verification of deep rock mass under the water-rock interaction. International Journal of Rock Mechanics and Mining Sciences. 2020;128:104250.

[23] Liu W, Zhang S. Creep constitutive model of rock based on strength time-dependent characteristics. Engineering Fracture Mechanics. 2024;298:109914.

[24] Liu W, Liu Q, Li J, Zhou H, Zhao C, Yang Y. An accelerated creep model for the rock downstream of a Xianglushan tunnel. Mechanics of Time-Dependent Materials. 2023;27(2):251–74.

[25] Lv Z, Liu C, Zhu C, Bai G, Qi H. Experimental study on a prediction model of the shrinkage and creep of recycled aggregate concrete. Applied Sciences. 2019;9(20):4322.

[26] Lyu C, Liu J, Ren Y, Liang C, Zhang Q. Study on long-term uniaxial compression creep mechanical behavior of rocksalt-mudstone combined body. International Journal of Damage Mechanics. 2022;31(2):275–93.

[27] Wang CP, Liu JF, Liang C, Jian L, Lu W, Yilin L. Creep constitutive model considering nonlinear creep degradation of fractured rock. International Journal of Mining Science and Technology. 2024;34(1):105–16.

[28] Wang J, Zhang Q, Song Z, Zhang Y. Creep properties and damage constitutive model of salt rock under uniaxial compression. International Journal of Damage Mechanics. 2020;29(6):902–22.

[29] Liu Y, Li Y, Mu J, Li H, Shen J. Modeling and analysis of creep in concrete containing supplementary cementitious materials based on machine learning. Construction and Building Materials. 2023;392:131911.

[30] Chen X, He C, Xu G, Wang S, Yun M. The creep behaviors of red sandstone in northern Yunnan and its fractional order damage modelling considering relaxation time effect. Bulletin of Engineering Geology and the Environment. 2024;83(8):313.

[31] Chen Y, Chen Q, Pan Y, Xiao P, Du X, Wang S, et al. A Chemical Damage Creep Model of Rock Considering the Influence of Triaxial Stress. Materials (Basel). 2022;15(21):7590. doi: 10.3390/ma15217590 36363180 PMC9655060

[32] Liu W, Zhang S, Zhao S, Xiang H. Intrinsic model of rock nonconstant damage creep based on fractal-order theory. Applied Mathematical Modelling. 2025;137:115681.

[33] Wu F, Yu Q, Liu C. Creep characteristics and constitutive model of bio-based concrete in aqueous environment. Construction and Building Materials. 2022;320:126213.

[34] Liu W, Zhou H, Zhang S, Jiang S. Constitutive model of concrete creep damage considering the deterioration of creep parameters. Construction and Building Materials. 2021;308:125047.

